# miR-22/KAT6B axis is a chemotherapeutic determiner via regulation of PI3k-Akt-NF-kB pathway in tongue squamous cell carcinoma

**DOI:** 10.1186/s13046-018-0834-z

**Published:** 2018-07-24

**Authors:** Yixue Gu, Hao Liu, Fangren Kong, Jiahui Ye, Xiaoting Jia, Zhijie Zhang, Nan Li, Jiang Yin, Guopei Zheng, Zhimin He

**Affiliations:** 1 Affiliated Cancer Hospital & Institute of Guangzhou Medical University, Guangzhou, China; 2 Guangzhou Key Laboratory of “Translational Medicine on Malignant Tumor Treatment”, Cancer Hospital and Cancer Research Institute of Guangzhou Medical University, Guangzhou, 510095 Guangdong China

**Keywords:** Tongue cancer, miR-22, KAT6B, NF-κB, p53, Chemotherapy response

## Abstract

**Background:**

Tongue squamous cell carcinoma (TSCC) is the most common oral cancer. Neoadjuvant systemic treatment before or after surgery for advanced TSCC is considered one of the most crucial factors in reducing mortality. However, the therapeutic benefits of chemotherapy are usually attenuated due to intrinsic and/or acquired drug resistance, and a large proportion of TSCC are resistant to chemotherapy, which may result in more aggressive tumor behavior and an even worse clinical outcome. Recently, the potential application of using miRNAs to predict therapeutic response to cancer treatment holds high promise, but miRNAs with predictive value remain to be identified and underlying mechanisms remain to be understood in TSCC.

**Methods:**

The expression of miR-22 in tissues from patients diagnosed with TSCC was analyzed using real-time PCR. The effects of miR-22 on cell proliferation and tumorigenesis in TSCC cells were analyzed by MTS assay, and flow cytometry. The tumor growth in vivo was observed in xenograft model. Luciferase reporter assay, real-time PCR and western blot were performed to validate a potential target of miR-22 in TC. The correlation between miR-22 expression and KAT6B expression, as well as the mechanisms by which miR-22 regulates PI3k-Akt-NF-kB pathway in TSCC were also addressed.

**Results:**

We found a strong correlation between miR-22 expression and chemosensitivity to cisplatin (CDDP) in TSCC patients. Ectopic overexpression of miR-22 enhanced TSCC cells apoptosis in response to CDDP in experimental models performed in vitro and in vivo. Moreover, we found that KAT6B is a direct functional target of miR-22. Ectopic expression of KAT6B attenuated the efficiency of miR-22 in TSCC cells upon CDDP treatment. Mechanistically, miR-22 overexpression or KAT6B knockdown inhibited PI3K/Akt/NF-κB signaling in TSCC cells, possibly via downregulating the activators of PI3K/Akt/NF-κB signaling, such as S100A8, PDGF and VEGF. Furthermore, the activation of miR-22 depended on the intensity of the stresses in the presence of p53 activation.

**Conclusions:**

Our findings define miR-22 as an intrinsic molecular switch that determines p53-dependent cellular fate through KAT6B/ PI3K-Akt/ NF-kB pathway.

## Background

Tongue cancer is the most common oral cancer, there were an estimated 12,060 new cases and 2030 deaths from tongue cancer in the United States in 2011 [1], in contrast there were an estimated 48,100 new cases and 22,100 deaths from tongue cancer in China in 2015 [2]. Tongue cancer is a rapidly progressing form of cancer that frequently metastasizes and has a poorer prognosis than carcinoma of other sites in the oral cavity. In the clinic, tongue cancer usually leads to malfunction of mastication, speech and deglutition. Neoadjuvant systemic treatment before or after surgery for advanced tongue cancer is considered one of the most crucial factors in reducing mortality. Chemotherapy mostly based on cisplatin (CDDP) is effective for reducing tumor size, inhibiting distant metastasis, preserving organ function, and prolonging patient survival [3]. However, the therapeutic benefits of chemotherapy are usually attenuated due to intrinsic and/or acquired drug resistance, and a large proportion of tongue cancers are resistant to chemotherapy, which may result in more aggressive tumor behavior and an even worse clinical outcome [4, 5]. Although the mechanisms responsible for chemotherapy resistance in cancer have being explored intensely for decades, the clinical causes of chemotherapy resistance are still very incompletely understood. In addition to the energy-dependent transporters that eject anti-cancer drugs from cells, multiple mechanisms, such as insensitivity to drug-induced apoptosis, increased DNA repair and induction of drug-detoxifying mechanisms, may also play important roles in chemotherapy resistance [6].

Biologically and clinically, a large number of studies have reported the important role of miRNAs in chemotherapy resistance [7]. miRNAs typically function in the post-transcriptional regulation of genes by binding to the 3′-untranslated region (3′UTR) of target messenger RNA (mRNA), mainly leading to translational repression or target mRNA degradation [8]. miRNAs have been shown to regulate many physiological and pathophysiological processes, such as development, differentiation, proliferation, stress response, metabolism and apoptosis, especially in cancer. miRNAs could function as both tumor suppressors and tumor promoters due to the diversity of miRNAs themselves [9]. With regard to cancer treatment, some studies have suggested that selected miRNAs may influence the cancer cell response to chemotherapy [10]. Specific miRNAs have shown altered expression in drug-resistant cancer cells. For example, miR-34a was downregulated in drug-resistant prostate cancer cells, and the ectopic expression of miR-34a resulted in growth inhibition and the sensitization of cells to camptothecin [11]; in addition, miR-200b expression was significantly downregulated in docetaxel-resistant NSCLC cells [12] . Furthermore, miRNAs also modulate the EMT (epithelial-mesenchymal transition) and the cancer stem cell program to influence the response to chemotherapy to cancer treatment [13, 14]. These reports strongly suggest an important role of miRNAs in cancer drug resistance, and further in-depth research is needed to fully understand this role and to find novel ways to regulate miRNAs to develop highly innovative treatment strategies.

With regard to the role of miRNA in tongue carcinogenesis and drug response, there are only a few reports. Wong et al. showed that miR-184 was overexpressed in tongue squamous cell carcinoma (TSCC), and that miR-184 inhibition reduced cell proliferation and induced apoptosis through c-Myc downregulation [15]. Li et al. reported that miR-21 was overexpressed in TSCC relative to the adjacent normal tissues, and miR-21 inhibition reduced TSCC growth and induced apoptosis in vitro and in vivo [16]. Qiu K and Colleagues found that down-expression of miR-22 can enhance cell growth and motility through inhibiting CD 147 in TSCC cells [17]. Recently, Sun et al. found that re-overexpression of miR-200b and miR-15b in cisplatin-resistant tongue cancer cells reduced BMI1 expression to sensitize the cells to chemotherapy via EMT modulation [18].

Disruption of the histone modification landscape is a common event in cancer cells [19, 20] leading to significant changes in chromatin structure and gene expression affecting oncogenes and tumor suppressor genes [21, 22]. Much effort has been devoted to analyzing the nucleosome of histone modifiers in search of key factors driven the origination and evolution of tumor. KAT6B (also named as MORF, MYST4), a histone acetyltransferase, have been implicated in leukemogenic and other tumorigenic processes [23]. KAT6B and and its homologous paralog MOZ (monocytic leukemic zinc-finger protein) have been shown to be involved in many biological processes, such as transcriptional regulation, DNA repair, the cell cycle and signal transduction [24]. However, the role of KAT6B in cancer chemosensitivity and chemoresistance has not been determined.

In the present study, we demonstrated a significant correlation between miR-22 expression and chemosensitivity to CDDP based regimen in tongue cancer patients. The increased chemosensitivity could be mediated by miR-22-dependent downregulation of KAT6B, resulting in inhibited NF-kB activity and increased apoptosis upon chemotherapy in tongue cancer cells. Furthermore, the activation of miR-22 depended on the intensity of the stresses in the presence of p53 activation. Therefore, miR-22 may be used as a predictive biomarker for CDDP based regimen and a potential therapeutic target in tongue cancer treatment.

## Methods

### Cell lines and cell culture

Human tongue cancer cell lines (CAL27 and SCC9), HCT 116 were obtained from and maintained as recommended by the American Type Culture Collection (ATCC, Manassas, VA, USA). HCT 116 p53^−/−^ was saved by our laboratory. Above cell lines were maintained in RPMI-1640 medium (Hyclone, Logan, UT, USA), supplemented with 10% fetal bovine serum (Hyclone) at 37 °C in a humidified atmosphere containing 5% CO_2_. The human embryonic kidney cell line (HEK-293 T) was cultured in DMEM (Gibco) with 10% fetal bovine serum. CDDP was purchased from Sigma-Aldrich (Sigma-Aldrich, US).

### Patient tissue specimens

All tongue cancer tissue specimens were collected via surgical resection from patients diagnosed between March 2007 and March 2013 at the Affiliated Tumor Hospital of Guangzhou Medical University (Guangzhou, Guangdong, China). Written informed consent was obtained from all study participants. The study protocol was approved by the Ethics Committee of Guangzhou Medical University. All eligible patients received three cycles of weekly CDDP-based regimen. Patients proceeded to surgery within 4 weeks of the last dose of chemotherapy. PCR was defined as the absence of invasive tumor cells in the final surgical tongue and ambient lymph node samples. Tumor tissue was obtained by a core biopsy prior to treatment and immediately stored at − 80 °C. Total RNA was extracted with Trizol reagent (Invitrogen, Carlsbad, CA, USA) following the manufacturer’s instructions. Overall survival was computed from the day of surgery to the day of death or of last follow-up.

### Real-time PCR for mature miRNAs and mRNAs

miRNAs from cultured cells were isolated and purified with the miRNA isolation system (Exiqon). cDNA was generated with the miScript II RT Kit (QIAGEN, Hilden, Germany), and quantitative real-time PCR (qRT-PCR) was performed by using the miScript SYBR Green PCR Kit (QIAGEN) following the manufacturer’s instructions. The miRNA sequence-specific RT-PCR primers and the endogenous control RNU6 were purchased from QIAGEN. The relative quantitative expression was calculated by normalizing the results with RNU6. The total RNA was extracted according to the Trizol protocol, and cDNAs from the mRNAs were synthesized with the first-strand synthesis system (Fermentas Life Science). The primers for C17orf91 and the selected mRNAs are shown in Table 1. Real-time PCR was carried out according to the standard protocol using an ABI 7500fast with SYBR Green detection (Fermentas SYBR green supermix). GAPDH was used as an internal control, and the qRT-PCR was repeated three times.

**Table 1:** Primers list

Gene	Forward primer (5′- to 3′)	Reverse primer (5′- to 3′)
C17orf91	CAAGTAGGGGAGGTGGGTTG	CTTCCTGTAGCCGCTAGGTG
EGF	TGTCCACGCAATGTGTCTGAA	CATTATCGGGTGAGGAACAACC
HGF	CAGCCCTGGAGTTCCATGATA	CCATTGCAGGTCATGCATTC
PDGF1	GTGAGGTTAGAGGAGCATTTGGA	CACATCTGGTTGGCTGCTTTAG
VEGF	GCCTCCGAAACCATGAACTTT	GACATCCATGAACTTCACCACTTC
IL-6	CCACTCACCTCTTCAGAACGAA	CAGTGCCTCTTTGCTGCTTTC
FGF2	AAGCGGCTGTACTGCAAAAAC	TCTCTCTTCTGCTTGAAGTTGTAGCT
CXCL3	GCCCCTGGCCACTGAACT	CAAGCTTTCTGCCCATTCTTG
S100A8	GCTGGAGAAAGCCTTGAACTCTAT	TGAGGACACTCGGTCTCTAGCA
S100A9	CAAAGAGCTGGTGCGAAAAGA	TCGAAGCTCAGCTGCTTGTC
IGF1	GCTCTTCAGTTCGTGTGTGGA	GCCTCCTTAGATCACAGCTCC
CXCL1	CGCCCAAACCGAAGTCATAG	CAGCCACCAGTGAGCTTCCT
CXCL3	GGGACAGCTGGAAAGGACTTAA	CAGGACTGAGCTATGTTTGATGAAA
CCL11	TCTGTGCCGACCCCAAGA	TGCATTGTAAGAAGGGAAAACAAA
GAPDH	ATTCCATGGCACCGTCAAGGCTGA	TTCTCCATGGTGGTGAAGACGCCA

### Cell transfection

The miR-22 expression plasmid pSilencer2.1-U6-miR-22 and an empty control vector were gifts from Dr. Xiuwu Zhang (Duke University Medical School, Durham, USA). The cells were transfected with these plasmids using Lipofectamine 2000 (Invitrogen) in accordance with the manufacturer’s instructions in 24-well plates. After 24 h, the cells were selected with 4 μg/ml puromycin for 2 weeks, and the individual stable clones were analyzed by qRT-PCR. Tongue cancer cells were transfected with pRNAT-U6.1/sh-KAT6B plasmids (Genscript, Nanjing, China) as described above, selected with 800 μg/ml G418 and validated by western blot. The anti-miR-22 oligonucleotides (QIAGEN) and the pBabe-IκBα or pEGFP-N1-WTp53 plasmids were transfected in 10-mm dishes with Lipofectamine 2000. The cells were harvested 72 h later, and the following experiments were performed.

### MTS assay

The Cell Titer 96® AQueous One Solution Cell Proliferation Assay kit (Promega, Madison, WI, USA) was used to determine the sensitivity of the cells to CDDP. In brief, the cells were seeded in 96-well plates at a density of 4 × 103 cells/well (0.20 ml/well) for 24 h before use. The culture medium was replaced with fresh medium containing CDDP with different concentrations for 72 h. Then, MTS (0.02 ml/well) was added. After 2 h of further incubation, the absorbance at 490 nm of each well was recorded on the Biotex ELX800. The growth rate was calculated as the ratio of the absorbance of the experimental well to that of the control well. The IC50 (the concentration of drug that results in 50% of control value) was also calculated.

### Cell apoptosis analysis

The cells (1 × 10^6^) were digested with a trypsin solution, then harvested and washed twice with cold PBS. The washed cells were rinsed twice with PBS, and re-suspended in a propidium iodine solution comprising containing 40 μg/mL propidium iodine and 100 lg/ mL RNaseA (Sigma-Aldrich) in PBS without calcium and magnesium, then incubated at 37 °C for 30 min in the dark. The stained cells were passed through a nylon mesh sieve to remove cell clumps, and then analyzed with FACScan flow cytometer and the CELL QUEST analysis software (Becton Dickinson, San Jose, CA, USA). The flow cytometry analysis was repeated three times.

### Western blot

The total proteins were extracted from the corresponding cells, loaded and separated on 10% SDS-PAGE and then transferred to PVDF membranes (Millipore, Billerica, MA, USA). The primary antibodies used for the analysis included mouse anti-KAT6B monoclonal Ab (Abnova, Taiwan, China); rabbit anti-phospho-PI3K p85 (Tyr458) polyclonal Ab, mouse anti-phospho-Akt (Thr308) monoclonal Ab, rabbit anti-PTEN monoclonal Ab and mouse anti-κBα (Ser32/36) monoclonal Ab (Cell Signaling Technology, Danvers, MA, USA); mouse anti-tubulin monoclonal Ab (Santa Cruz, Dallas, Texas, USA).

### Luciferase reporter assay

Two single strands of the wild type 3’UTR with the miR-22 binding site and two single strands of the mutant type with seven bases deleted in the miR-22 binding site (as a mutant control) of KAT6B were synthesized with restriction sites for SpeI and HindIII located at both ends of the oligonucleotides for further cloning. The single-strand DNA sequences were as follows: wild type 3’UTR of KAT6B (sense: 5’-CTAGTCAGATTTCTTTGGGGAAAAAAGGCAGCTTTCTGTTTTATAAATGCAGACTTCTGA-3′; antisense: 5’-AGCTTCAGAAGTCTGCATTTATAAAACAGAAAGCTGCCTTTTTTCCCCAAAGAAATCTGA-3′) and the mutated type 3’UTR of KAT6B (sense: 5’-CTAGTCAGATTTCTTTGGGGAAAAAA-------TTCTGTTTTATAAATGCAGACTTCTGA-3′; antisense: 5’-AGCTTCAGAAGTCTGCATTTATAAAACAGAA-------TTTTTTCCCCAAAGAAATCTGA-3′). The corresponding sense and antisense strands were annealed and subsequently cloned into the pMir-Report plasmid downstream of the firefly luciferase reporter gene. The cells were seeded in 96 well-plates and co-transfected with the pMir-Report luciferase vector, the pRL-TK Renilla luciferase vector and the miR-22 expression vector. After 48 h, the luciferase activities were determined using a Dual-Luciferase Reporter Assay System (Promega) where the Renilla luciferase activity was used as an internal control and the firefly luciferase activity was calculated as the mean ± SD after being normalized relative to the Renilla luciferase activity. To identify the promoter of miR-22, a 2-kb region upstream of the miR-22 precursor starting site was cloned into the pGL4-reporter vector upstream of the luciferase gene and the reporter vector with site B binding site deletion as mutant was also constructed, and the reporter assay was performed as described above.

### NF-κB activity

The cells were seeded in 96-well plates and co-transfected with the NF-κB reporter plasmid or the corresponding control, the pRL-TK Renilla luciferase plasmid and/or the pBabe-IκBα plasmid. Then, the luciferase activities were determined using the Dual-Luciferase Reporter Assay System (Promega).

### Caspase 3 activity assay

Caspase 3 activity was determined with a caspase 3 activity kit (Beyotime, China) through the cleavage of a colorless substrate specific for caspase 3 [Ac-DEVD-p-nitroaniline (pNA)], releasing the chromophore pNA. The assays were carried out according to the manufacturer’s instructions39.

### In vivo tumorigenesis assays

BALB/c-nude mice (female, 3–4 weeks of age, 18-20 g) were purchased from the Center of Experimental Animal of Guangdong province. All experimental procedures were approved by the Institutional Animal Care and Use Committee of Guangzhou Medical University. The BALB/c nude mice were randomly divided into 2 groups (each group contain 10 mice). CAL27 stably expressing miR-22 or the control cells (5 × 10^6^/site) were inoculated subcutaneously in the dorsal flanks of nude mice and developed into solid tumors in 7–10 days after injection. When the maximum tumor diameter exceeds 6um, each group were respectively treatment with 2 mg/kg cisplatin or equal volumes saline by intraperitoneally injection every other day for 4 weeks.

In addition, an in vivo loss-of-function study by intravenously administering miR-22 siRNA to xenograft tumor mouse model was performed. CAL27 cells (5 × 10^6^/site) were subcutaneously injected of into immunodeficient mice. All mice inoculated with CAL27 cells developed primary tumors, which were readily visualized and the maximum tumor diameter exceeds 6um in 8–10 days. We treated these mice intravenously with in vivo -jetPEI-formulated mir-22 inhibitor for 4 weeks. Separate groups of animals bearing CAL27 xenograft tumors were treated with the same doses of in vivo -jetPEI-formulated tRNA/MSA as controls (tRNA/mir-22 or control tRNA scaffold were conducted as described recently [25–27]). Meanwhile, 2 mg/kg cisplatin or equal volumes saline was intraperitoneally injected every other day for 4 weeks. Tumor sizes were measured with caliper every 3 days and calculated by the formula V = (L × W^2^)/2. Thirty days after tumor implantation, the mice were sacrificed; the subcutaneous tumors were removed and weighed. Tumors were fixed in formalin and embedded in paraffin using the routine method. Serial 6.0 μm sections were cut and subjected to H&E stained with Mayer’s hematoxylin solution.

### ChIP assay

The ChIP assay was performed using the EZ-CHIPTM chromatin immunoprecipitation kit (Millipore). In brief, chromatin proteins were crosslinked to the DNA by addition of formaldehyde to the culture medium to a final concentration of 1%. After 10 min of incubation at room temperature, the cells were washed and scraped off in ice-cold PBS containing a Protease Inhibitor Cocktail II. The cells were pelleted and then resuspended in Lysis Buffer containing Protease Inhibitor Cocktail II. The resulting lysate was subjected to sonication to reduce the size of DNA to 200–1000 bp. The sample was centrifuged to remove the cell debris and diluted 10-fold in ChIP dilution buffer containing Protease Inhibitor Cocktail II. An aliquot of 5 μl of the supernatant was removed as the “Input” and saved at 4 °C. Then, 5 μg of the anti-RNA Polymerase antibody (as positive control), the normal rabbit IgG (as a negative control) (provided by kit) or the anti-p53 antibody were added to the chromatin solution and incubated overnight at 4 °C with rotation. After antibody incubation, the protein G agarose was added and incubated at 4 °C with rotation for 2 h. The protein/DNA complexes were washed with Wash Buffers four times and eluted with ChIP Elution Buffer, and the cross-links were reversed to free the DNA by the addition of 5 M NaCl and incubation at 65 °C for 4 h. The DNA was purified according to the manufacturer’s instructions, and 50 μl of the DNA from each treatment was obtained. A volume of 0.2 μl of DNA from each group was used as a template for PCR. The primers for the miR-22 promoter with p53 binding sites were as follows: Sense: 5’-GCGGTGCCGGGGCCTTAT-3′, Antisense: 5’-GGTGCAGAGGTGACCTTCTCTC-3′ (for site A, product: 148 bp); sense: 5’-CAGGGGAAGGAAGATACACAAAGT-3′, antisense: 5’-GTTCCAACGCATGAATCAGCAG-3′, (for site B, product: 173 bp); sense: 5’ ACTGAGTGTCAGCACGGACCC-3′, Antisense: 5’-TTCCAAGTTCCAAGCCCCG-3′(for site C, product: 149 bp). The primers for the positive and negative controls or “no DNA” were specific for the human GAPDH gene: Sense, 5’-TACTAGCGGTTTTACGGGCG-3′, Antisense, 5’-TCGAACAGGAGGAGCAGAGAGCGA-3′, (product: 166 bp). The PCR conditions were as follows: 1 cycle of 95 °C for 5 min; 32 cycles of 95 °C for 20 s, 59 °C for 30 s, and 72 °C 30 s; and 1 cycle of 72 °C for 10 min. The PCR samples were resolved by electrophoresis in a 2% agarose gel and stained with ethidium bromide.

### Immunohistochemistry (IHC) staining

IHC staining was performed according to the protocol previously described [28]. In brief, the tissue sections were deparaffinized with dimethylbenzene and then rehydrated via a graded alcohol series. Endogenous peroxidase activity was blocked with 0.3% hydrogen peroxide for 15 min. The slides were boiled in tris(hydroxymethyl) aminomethane-EDTA buffer (pH 8.0) in a microwave for 30 min to retrieve antigen. Nonspecific antigens were blocked with 10% normal goat serum for 20 min. Then, the slides were incubated with first antibody (anti-KAT6B monoclonal Ab (Abnova, Taiwan, China, 1:200 dilution); mouse anti-phospho-Akt (Thr308) monoclonal Ab, 1:200 dilution, Abnova) overnight at 4 °C in a moist chamber. The controls were treated by replacing the primary antibody with normal goat serum. On the second day, the slides were sequentially incubated with biotinylated rabbit anti-mouse antibody, streptavidin-peroxidase conjugate and 3′-3’diaminobenzidine. The immunoreactivity scoring criteria are as described previously [28]. The staining result for each section was the average scores decided by two pathologists.

### Statistical analysis

Each experiment was repeated at least three times. The statistical analysis was carried out using SPSS 16.0. The Student’s t-test was chosen to analyze the significant differences. The results were presented as the mean ± SD. *P* < 0.05 was considered statistically significant.

## Results

### Expression level of miR-22 correlates with treatment response and disease-free survival

**Fig. 1:**
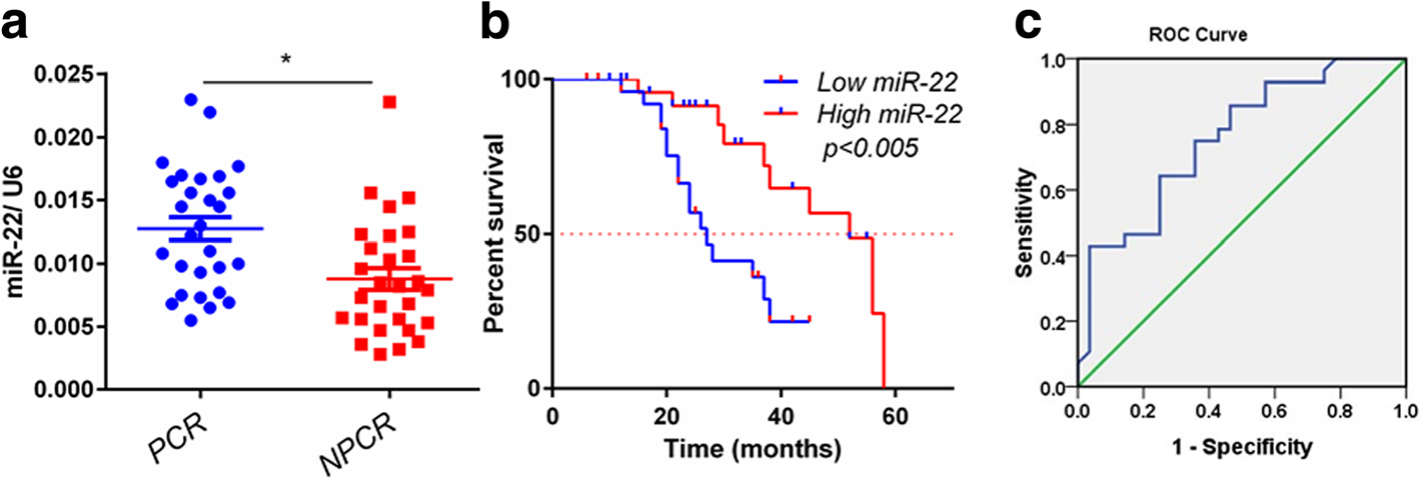
High expression level of miR-22 correlates with pathological complete response (pCR) and higher disease-free survival (DFS). **a**, Expression levels of miR-22 in tongue cancer patients (*n* = 56, 28 pCR vs 28 non-pCR), who received CDDP regimen chemotherapy, were quantitated by real-time PCR. RNU6 was used as an internal control. Data are mean ± s.e.m. **b**, Kaplan–Merier curves of 56 tongue cancer patients after stratification by the level of miR-22 were used for depicting DFS. **c**, Receiver operating characteristic (ROC) curve of 56 oral cancer patients’ level of miR-22 was used for analyzing the area under the curve (AUC) value

To validate the association between miR-22 and response to CDDP based chemotherapy regimen, an independent set of 56 patients (28 pCR patients and 28 non-pCR patients) was analyzed by using quantitative RT-PCR. The clinicopathological features of this validation set are listed in Table 2. Consistently, we found the expression level of miR-22 was significantly higher in tumors from pCR patients than that in non-pCR patients (*p* = 0.015) (Fig. 1a), confirming that the higher expression of miR-22 is associated with a better response to CDDP based chemotherapy regimen. Furthermore, analyzing the disease-free survival (DFS) of these 56 tongue cancer patients after stratification by the level of miR-22 revealed that patients with higher miR-22 expression level have significantly longer DFS (*P* < 0.005) (Fig. 1b). To evaluate this predictive value, we used the receiver operating characteristics (ROC) curve to analyze the sensitivity and specificity of miR-22. The area under the curve (AUC) was 0.827 (confidence interval = 0.733–0.926; *P* < 0.0001), which indicated high accuracy of predictive value. The sensitivity and specificity were 88.3 and 65.6%, respectively (Fig. 1c). These findings suggest that increased miR-22 expression may be responsible for high sensitivity to CDDP based chemotherapy and predict pCR in tongue cancer patients.

**Table 2:** Clinical information and demographics of the 56 patients included in the study

Characteristics	pCR		n-pCR		*P*-value
	No	%	No	%	
Age (years)					0.585
> 50	10	17.9	13	23.2	
≤50	18	32.1	15	26.8	
sex					0.851
Male	16	28.6	17	30.4	
female	12	21.4	11	19.6	
Node metastasis					0.412
No	13	23.2	9	16.1	
N1–2	15	26.8	19	33.9	
Clinical stage					0.296
II- IIIA	25	44.6	21	37.5	
IIIB- IV	3	5.4	7	12.5	
Status					0.043*
Survival	13	19.6	5	8.9	
Death	15	30.4	23	41.1	

Fisher’s exact test was used to analyze the categorical variables. *P < 0.05

### miR-22 increases chemosensitivity to CDDP in tongue cancer cells

**Fig. 2:**
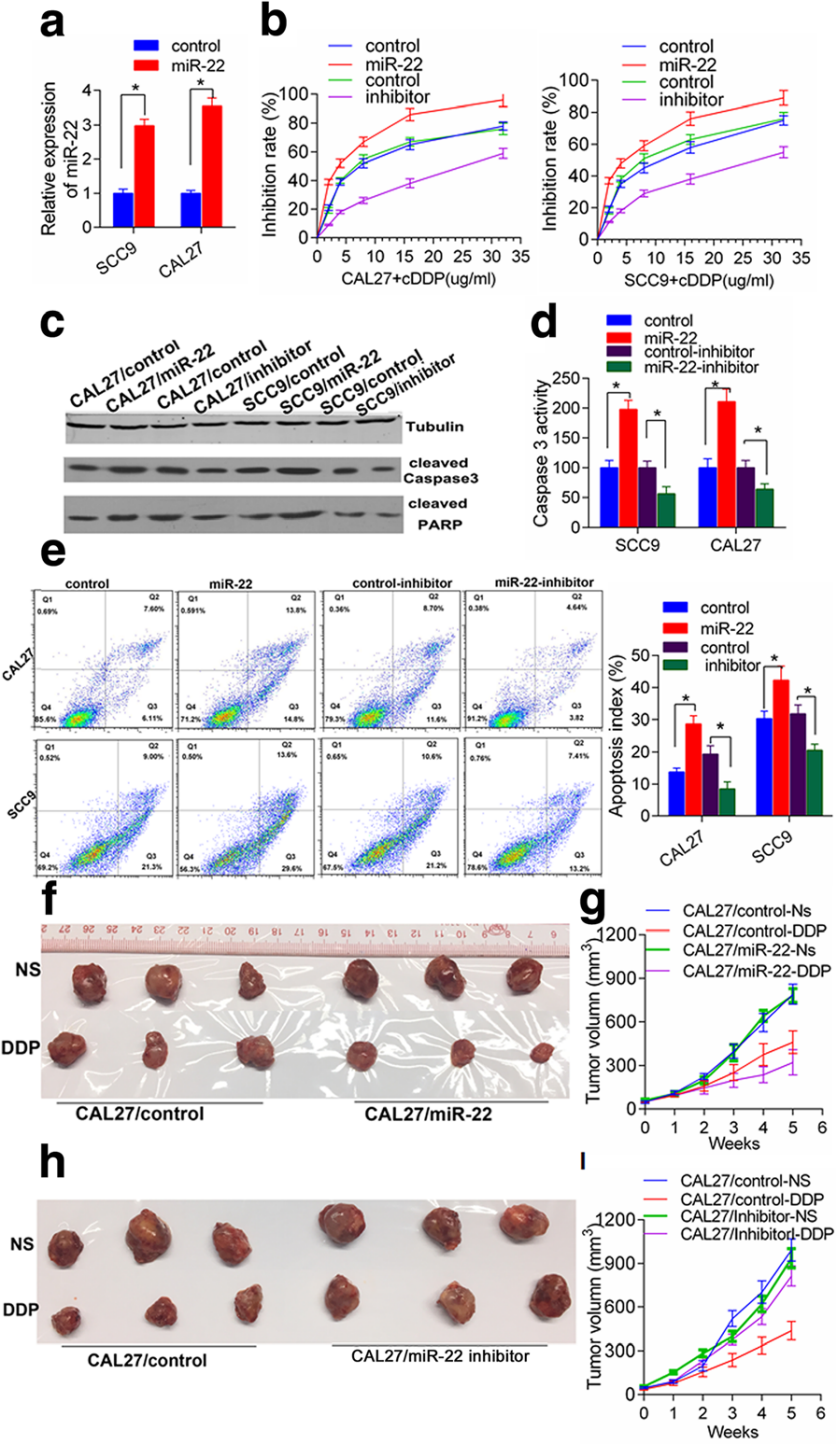
MiR-22 increases chemosensitivity to CDDP in tongue cancer cells. **a** The miR-22 expression in the selected clones indicated that miR-22 overexpressing cell lines were successfully established; **b**, dose survival curve was plotted from the MTS assay results collected from three independent experiments, showing that miR-22 enhanced the effect of cDDP on oral cancer cells, and miR-22 inhibition promoted the resistance of both cell lines to cDDP. **c** and **d**, indicated cells were incubated in the presence of 4 μg/ml CDDP for 24 h and cleaved caspase 3 or PARP was detected by immunoblotting. **e**, cells were treated with 4 μg/ml CDDP for 24 h; apoptotic cells were determined by FACS. Quantification of apoptotic cells using 3 independent FACS experiments. Data show mean with SD. *P < 0.05. **f**, **g**, Mice transplanted with CAL-27/miR-22 stable cells (n = 5) or CAL-27/vector control cells (n = 5) were mock treated or treated with CDDP as shown. Tumor growth curve-based tumor volume measure was shown as figure **g**. *P < 0.05. (**h**-**i**), Separate groups of animals bearing CAL27 xenograft tumors were treated with Vivo -jetPEI-formulated mir-22 inhibitor or controls accompanied with 2 mg/kg CDDP or equal volume saline as shown. Xenograft tumor growth was monitored and growth curve was mapped in figure **i**. *P < 0.05

To investigate whether miR-22 has a direct function in the response to chemotherapy in TSCC cells, we used a gain- or loss-of-function approach in SCC9 and CAL27 cells. As shown in Fig. 2a, stable miR-22 overexpressing cells were established by extraneous transfection with pSilencer2.1-U6-miR-22 plasmid. MiR-22 overexpression significantly enhanced the sensitivity of SCC9 and CAL27 cells to cDDP (Fig. 2b). Consistently, modulating miR-22 level by transfecting miR-22 inhibitor enhanced TSCC cells survival upon cDDP treatment (Fig. 2b). These results collectively support that increasing expression of miR-22 might enhance drug sensitivity to cDDP in tongue cancer cells. Furthermore, Overexpression of miR-22 in SCC9 and CAL27 cells markedly increased the cleavage of Caspase 3 and PARP1 in response to cDDP treatment, suggesting an enhanced apoptosis induced miR-22 upon drug treatment (Fig. 2c and d). We next measured the effect of miR-22 on apoptosis using propidium iodide and Annexin V apoptosis assay. The data indicated miR-22 transfection resulted in a significant increase in the percentages of cells undergoing apoptosis; whereas miR-22 inhibition noticeably prevented apoptosis of CAL27 and SCC9 cells (Fig. 2e). In addition, we validated whether miR-22 could play a role in tumor sensitivity to cDDP in vivo. MiR-22 overexpressing CAL27 cells and related control cells were transplanted into nude mice subcutaneously and developed into solid tumors in 7–10 days, and then 2 mg/kg cisplatin or equal volume saline were intraperitoneally injected every other day for 4 weeks. The tumor growth curves and tumor inhibited rate histogram were mapped. As shown in Fig. 2f and g, the tumor volume of miR-22-overexpressing xenografts decreased to a greater extent than that of control xenografts upon cDDP treatment, indicating a chemo-sensitizing effect of miR-22. Meanwhile, we performed an in vivo loss-of-function study by intravenously administering miR-22 siRNA to xenograft tumor mouse model through subcutaneous injection of CAL27 cells into immunodeficient mice, and evaluated the effectiveness of miR-22 inhibitor to sensitivity to cDDP in vivo. We treated these mice intravenously with in vivo -jetPEI-formulated tRNA/mir-22 accompanied with 2 mg/kg CDDP or equal volume saline for 4 weeks. Compared with the same doses vehicle, tRNA/mir-22 treatment showed a remarkable suppression of the chemosensitivity of xenografts tumors (*P* < 0.01, Fig. 2h, i). These results indicate that administration of biological miR-22 is effective to enhance tongue cancer chemotherapy sensitivity in vivo.

### miR-22 enhances the sensitivity of TSCC cells to cisplatin by targeting KAT6B expression

**Fig. 3:**
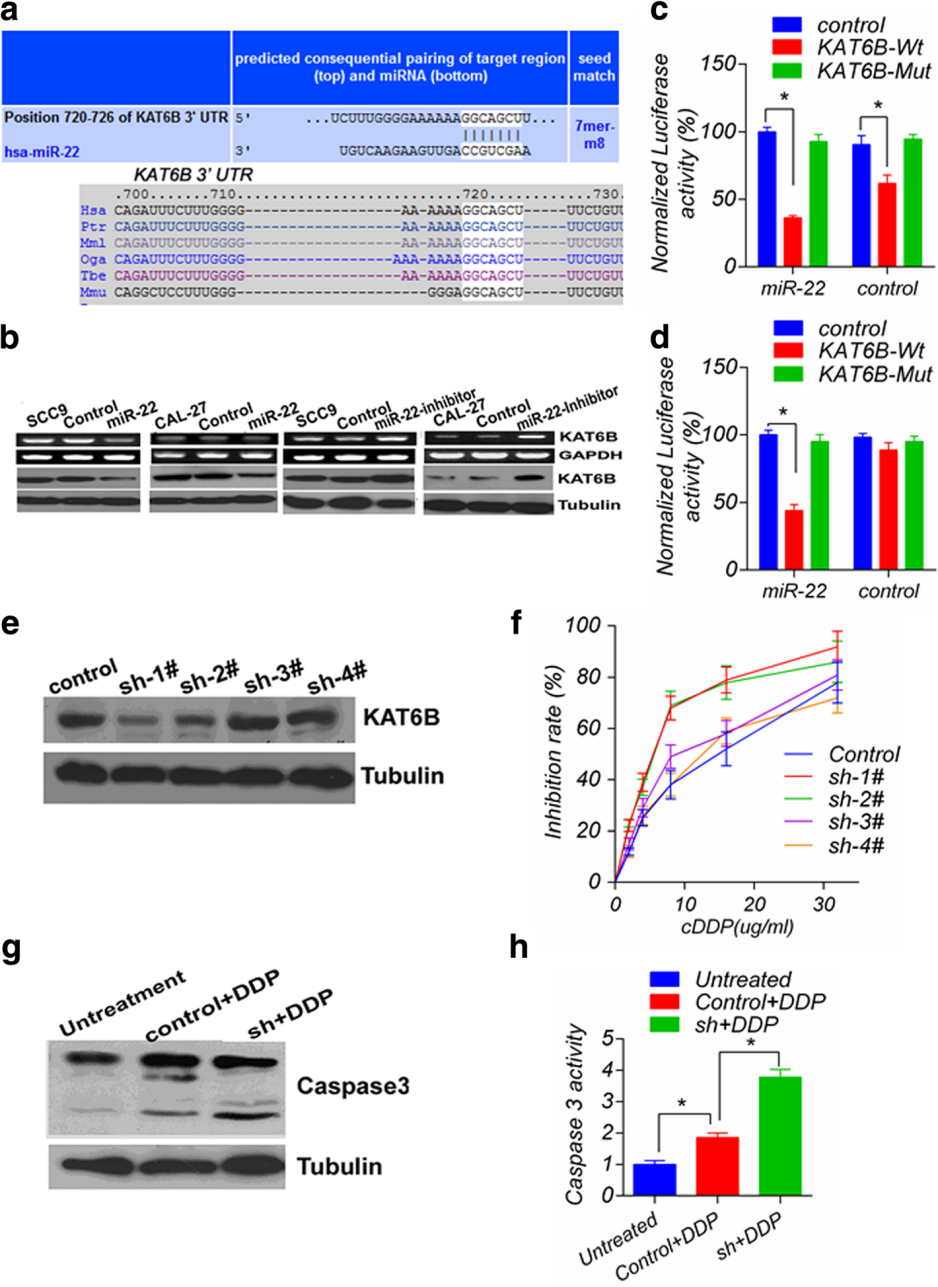
Expression of the miR-22 target KAT6B and KAT6B knockdown sensitized tongue cancer cells to CDDP: **a** Schematic of the predicted miR-22 site in the human KAT6B 3′UTR, which is broadly conserved among vertebrates; **b** Inverse relationship between miR-22 and KAT6B mRNA and protein levels is shown; **c** and **d** miR-22 suppressed the activity of the luciferase gene linked to the 3′UTR of KAT6B, and a Renilla luciferase reporter was used for normalization. The data were obtained from three independent experiments. The mean of the results from CAl27 (**c**) transfected with the pMir-control and 293 T cells (**d**) transfected with the pMir-control and miR-22 were set as 100%, respectively. * *p* < 0.05. **e** The KAT6B expression change mediated by sh-KAT6B showed that sh-1# significantly reduced the KAT6B protein level; **f** Dose-inhibition rate curves were plotted from the MTS assay results collected from three independent experiments, showing that sh-1# significantly decreased cell viability upon CDDP treatment, with a left-shifted IC50 curve; **g** Western blot analysis revealed that sh-KAT6B enhanced CDDP-induced caspase 3 activation; **h** The effect of sh-KAT6B on CDDP-induced caspase3 activation in SCC9 cells. The relative activation of caspase3 was calculated from the average of three experiments. Each value is expressed as the ratio of the caspase3 activation level to the untreated level, and the untreated level was set as 1. *versus untreated, *p* < 0.05

To elucidate the underlying mechanisms promoting chemosensitivity in human tongue cancer cells by miR-22, we used the public database TargetScan (http://www.targetscan.org) to predict potential targets of miR-22, and KAT6B (MYST4) with a critically conserved binding site was selected for further identification (Fig. 3a). Notably, the endogenous overexpression of miR-22 in CAL27 and SCC9 cells was accompanied with decreased expression of KAT6B mRNA and protein (Fig. 3b). In contrast, depression miR-22 by transfecting miR-22 inhibitor increased the expression of KAT6B. To assess whether KAT6B is a direct target of miR-22, a luciferase reporter vector with a putative KAT6B 3’UTR target site for miR-22 downstream of the luciferase gene (pMir-KAT6B-Wt) and a mutant version with a deletion of 7 bp in the seed region was constructed (pMir-KAT6B-Mut). As shown in Fig. 3c, d, the luciferase reporter assay performed in CAL27 and HEK293T cells showed that miR-22 reduced the luciferase activity of the vector with the wild-type KAT6B 3’UTR, but the mutant version abrogated the repressive ability of miR-22. These results strongly demonstrated the specificity of miR-22 targeting KAT6B. To investigate the function of KAT6B, CAL27 cells were transfected with pRNAT-U6.1/sh-KAT6B to knockdown KAT6B, and sh-1# markedly decreased KAT6B protein expression (Fig. 3e). Therefore, sh-1# was selected for further study. A negative relationship between KAT6B and the response to CDDP was also observed (Fig. 3f). In addition, sh-1# promoted the effect of 10 mg/L CDDP on caspase3 activation in CAL27 cells. As shown, caspase3 cleavage was detected (Fig. 3g) and caspase3 activity increased (Fig. 3h).

### Expression of KAT6B inversely correlates with miR-22 and correlated to poor DFS progression in tongue cancer patients

**Fig. 4:**
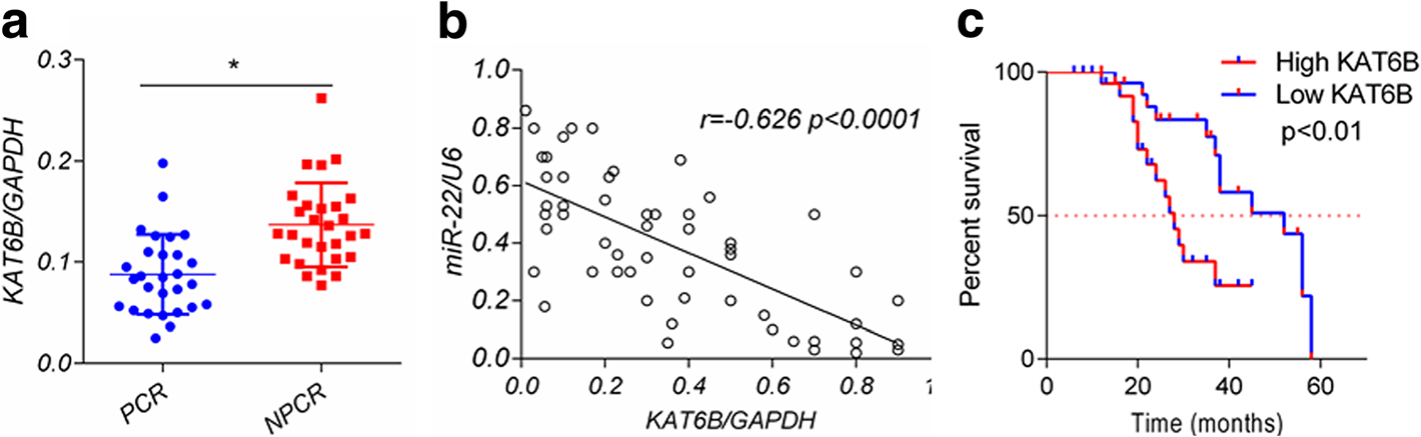
KAT6B inversely correlates with miR-22 and correlated to poor DFS progression in tongue cancer patients. **a**, Expression levels of KAT6B in 56 tongue cancer patients who received cDDP based regimen neoadjuvant chemotherapy were analyzed by quantitative PCR. GAPDH was used as an internal control. Data were shown as mean ± SD. **b**, Spearman rank test of 56 tongue cancer patients was used for depicting the correlation between KAT6B and miR-22. **c**, High KAT6B level correlated to poor DFS in tongue cancer patients received chemotherapy

Given the regulatory relationship between miR-22 and KAT6B identified in our cell culture studies, we focused whether the miR-22/KAT6B might associate with the prognosis of tongue cancer patients. The expression levels of miR-22 and KAT6B were evaluated in clinical samples of tongue cancer patients by Q-PCR. We found that the expression of KAT6B in pCR patient tumors was significantly lower than that in non-pCR patients (*P* < 0.05, Fig. 4a). Moreover, expression of KAT6B presented a negative correlation with miR-22 level in these 56 patients (*r* = − 0.626, *P* < 0.0001) (Fig. 4b). Additionally, we found high KAT6B level was associated with poor DFS in these 56 patients (Fig. 4c). Collectively, these data indicate that the chemo-sensitizing effect of miR-22 in tongue cancer may depend on KAT6B downregulation.

### miR-22/KAT6B axis enhances chemosensitivity to cDDP by inhibiting PI3K/Akt/NF-κB activity in tongue cancer cells

**Fig. 5:**
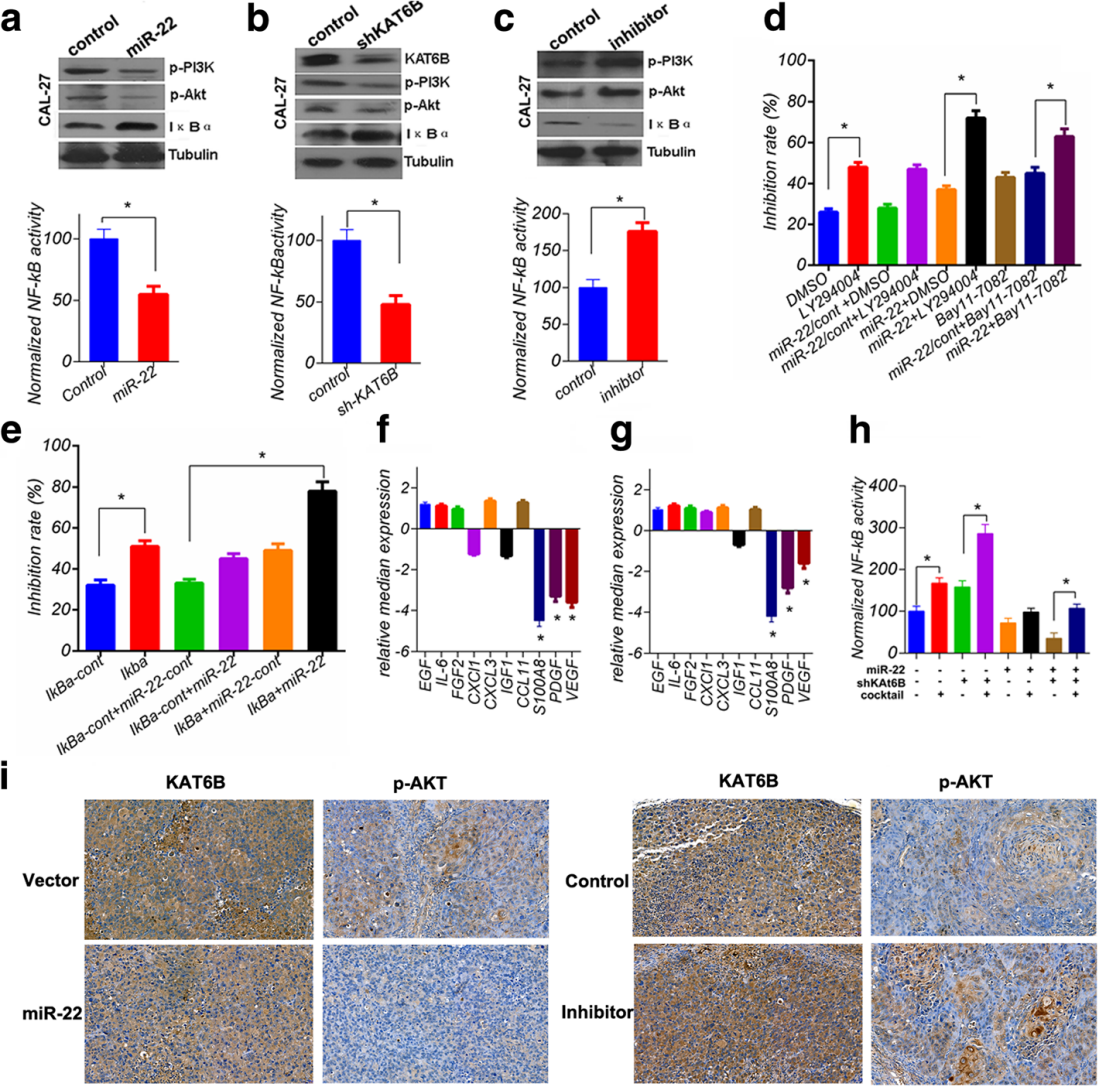
miR-22/KAT6B determines cellular fate via Akt-NF-kB pathway. **a** and **b** miR-22 overexpression or KAT6B knockdown inhibited PI3K/Akt/NF-κB activity in CAL27 cells. In contrast, miR-22 inhibition further promoted PI3K/Akt/NF-κB activity (**c**). **d**-**e**, The inhibition rate was calculated 72 h later after the initial treatment of CAL27 cells. The data were obtained from three independent experiments in triplicate versus no treatment or control treatment; (**f**-**g**) qRT-PCR showed that miR-22 overexpression or KAT6B knockdown reduced S100A8, PDGF and VEGF expression. **h**, mixed cytokines S100A8, PDGF and VEGF attenuated inhibition of NF-kB activity in miR-22-overexpressing or shKAT6b tongue cancer cells. The data shown here are the mean ± SD from three independent experiments conducted in triplicate, versus control *p < 0.05. **i**, Ectopic xenograft tissue obtained from the previous animal experiment, which were fixed by formaldehyde, paraffin embedded, continuous sliced and tested for the immunization of KAT6B and AKT signaling molecules. The representative images are shown

To explore the signal transduction pathway responsible for the up-regulation of chemosensitivity promoter-miR-22 in CAL27 cells, the activity of the predominant oncogenic pathway, PI3K/Akt/NF-κB, was examined. The results shown that both miR-22 overexpression and KAT6B knockdown inhibited PI3K/Akt/NF-κB activation, respectively (Fig. 5a, b), with no difference in PTEN expression (data not shown). Whereas, miR-22 inhibition further promoted PI3K/Akt/NF-κB activity (Fig. 5c). These data implied miR-22 enhanced chemosensitivity to cDDP by inhibiting PI3K/Akt/NF-κB activity in tongue cancer cells. To observe the exact role of PI3K/Akt/NF-κB and miR-22 in drug resistance, a series of treatments was applied in CAL27 cells and the drug sensitivity was measured. Treatment with 10 mg/L CDDP, 40 μM LY294004, 10 μM Bay11–7082 and IκBα expression significantly enhanced the effect of CDDP. The effect of PI3K/Akt/NF-κB inhibition combined with miR-22 overexpression was much stronger than either alone (Fig. 5d, e). To address how miR-22 or KAT6B regulated PI3K/Akt/NF-κB activity, we sought to examine the expression of some cytokines in miR-22 overexpression or KAT6B knockdown in CAL27 cells. As shown in Fig. 5f and g, S100A8, PDGF and VEGF were significantly downregulated in forementioned cell lines. Interestingly, these secretory cytokines had been proved to activate PI3K/Akt / NF-κB pathway in previous researches [29–31]. To confirm whether miR-22/KAT6B potentially activate PI3K/Akt/NF-κB signal pathway through these secretory cytokines, indicated concentration cytokines S100A8, PDGF and VEGF were mixed as a cocktail and added into cell culture medium, then the NF-κB activity was evaluated. The data indicated that mixed cytokines attenuated inhibition of NF-kB activity in miR-22-overexpressing or shKAT6b tongue cancer cells (Fig. 5h). These results suggested that S100A8, PDGF and VEGF may be the targets or the downstream of KAT6B, which potentially activate PI3K/Akt/NF-κB signal pathway. In addition, to further verify that miR-22/KAT6B functions in the chemotherapy resistance through the AKT-NF-κB pathway, we selected the heterotopic tumor tissue obtained from the previous animal experiment, which were fixed by formaldehyde, paraffin embedded, continuous sliced and tested for the immunization of KAT6B and AKT signaling molecules. The results indicated that miR-22 does inhibit the expression of KAT6B and the phosphorylation of AKT. On the contrary, reduced miR-22 can activate the AKT signaling pathway, these data were shown in Fig. 5i.

### Intensity of stress determines the pattern of miR-22 transcriptional activation

**Fig. 6:**
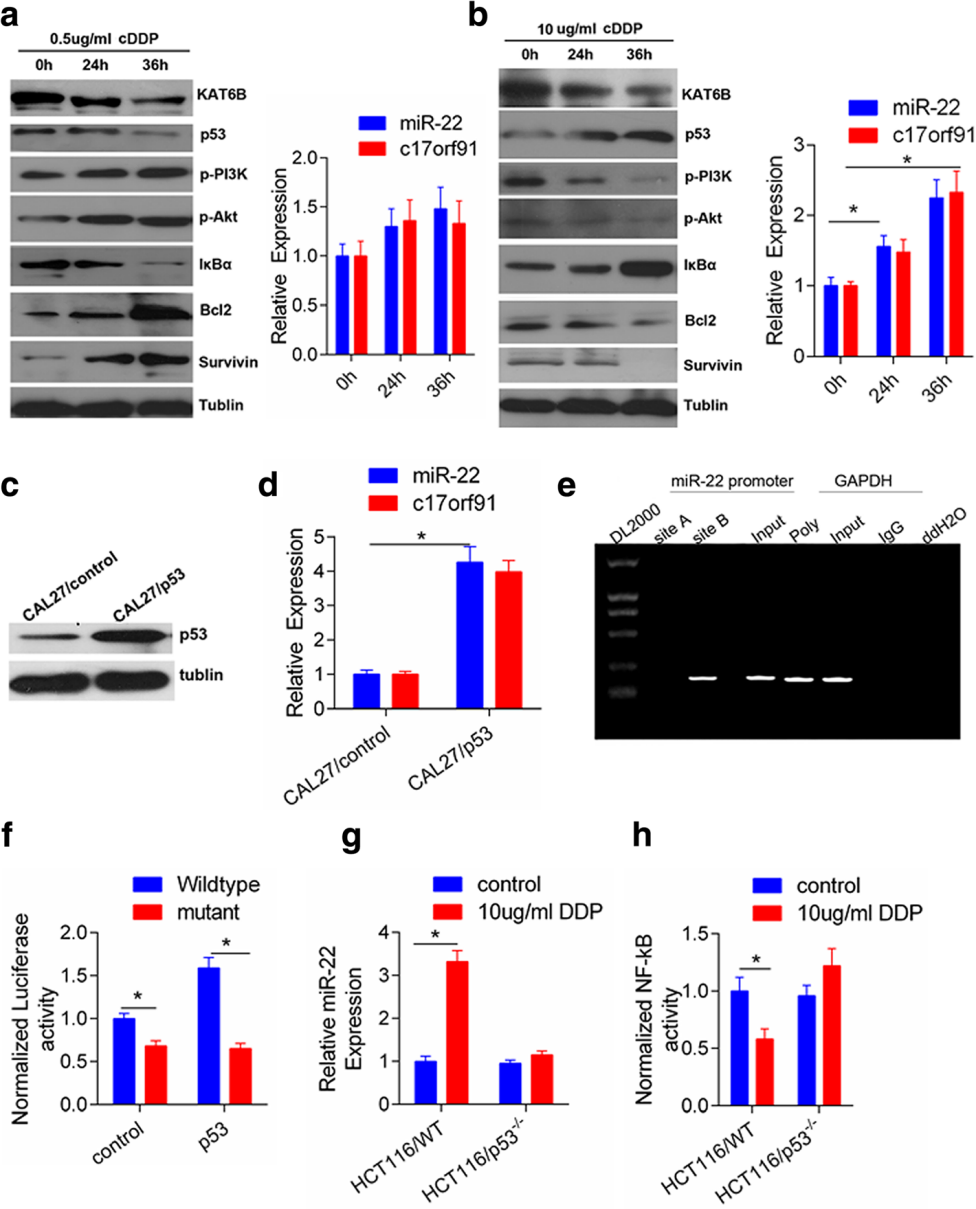
Stress-induced miR-22 transcriptional expression patterns depended on the p53 activation. **a** Time-dependent changes of PI3K/Akt/NF-κB pathway molecules and p53 after exposure to low doses (0.5μg/ml) of CDDP; accordingly, there were no significant difference on increments of expression of miR-22 and C17orf91 quantified by qRT-PCR. **b**, Time-dependent changes of PI3K/Akt/NF-κB pathway molecules and p53 after exposure to higher doses (10μg/ml) of CDDP; accordingly, significant difference was observed on increments of expression of miR-22 and C17orf91 quantified by qRT-PCR, versus no treatment, *p < 0.05. **c** and **d** Ectopic overexpression of p53 in CAL-27 cells enhanced the expression of miR-22 and C17orf91 at the transcriptional level, versus control, *p < 0.05. **e** Chromatin immunoprecipitation assays identified p53 binding sites within the putative promoter. PCR reaction products from the miR-22 promoter group, site A and site B represent p53 immunoprecipitation, and the input represent DNA directly after lysis. In the GAPDH group, the PCR product from the RNA Polymerase antibody represents positive control, and the PCR from IgG and ddH2O (no DNA) represent the negative controls; **f** the activity of the putative promoter of miR-22 was higher in CAL27 cells with endogenous miR-22 overexpression. **g** and **h**, HCT116 WT and HCT116 p53^−/−^ cells were treated with control or CDDP (10 μg/ml). Relative fold induction of miR-22 expression and NF-kB activity were shown as mean ± s.d. *P < 0.05

Due to the drug resistance acquired through exposure to treatment stress, to investigate the expression pattern of miR-22 on stress, the kinetics of miR-22 expression were examined when treating CAL27 cells with different doses of CDDP. There was no significant cell death observed in CAL27 cells treated with 0.5 mg/L CDDP, and the cell survival promoting pathway was triggered. As shown in Fig. 6a, the p-PI3K, p-Akt and NF-κB targets Bcl-2 and Survivin increased and the IκBα protein level decreased in a time-dependent manner, without significant a change in miR-22 and C17orf91 expression at an early state (Fig. 6a). The miR-22 primary transcript is located in the 5′-untranslated region of C17orf91 and shares the same promoter/enhancer [32]. Therefore, we also measured the change in expression of C17orf91 under the conditions described above. In contrast, upon exposure to lethal-dose 10 mg/L CDDP, the p-PI3K, p-Akt and NF-κB targets Bcl-2 and Survivin decreased. Whereas, IκBα expression were increased (Fig. 6b). These data indicated different intensities of stress trigger different dominant signaling pathways to determine different cell fates. Due to overexpression of miR-22 in CAL27 and SCC9 cells markedly increased the cleavage of Caspase 3 and PARP1 in response to CDDP treatment, suggesting an enhanced apoptosis upon drug treatment (Fig. 2c and d). The tumor suppressor p53 activates a key function that directs cells to undergo apoptosis or senescence in response to cellular stress [33, 34]. Therefore, we asked whether p53 orchestrated chemosensitivity to CDDP with miR-22/KAT6B in tongue cancer cells. Interestingly, we found that the expression of p53 is significantly increased upon higher doses but not low doses CDDP treatment, accompanied by increased expression of miR-22 and C17orf91 (Fig. 6a, b). To further address whether unregulated miR-22 depends on p53 activation in CAL27 cells, we performed the transient transfection of CAL27 cells with pEGFP-N1-WTP5 and the subsequent p53 protein increase was accompanied by an increase in miR-22 and C17orf91 expression (Fig. 6c, d). In addition, to validate the direct association of p53 with the miR-22 promoter, we performed a ChIP assay in CAL27 cells for all the putative p53binding sites within 2-kb region. The ChIP results revealed that p53 most significantly bound to site B within the miR-22 promoter (Fig. 6e). The luciferase expression driven by the miR-22 promoter with site B was significantly higher than site mutant in HEK-293 T cells (Fig. 6f). To further confirm whether p53 can enhance miR-22 activity, we examined HCT116 WT and HCT116 p53^−/−^ cells, and treated these cells with CDDP. Expression of miR-22 and C17orf91were significantly activated in HCT116 WT cells, but not in p53^−/−^ cells, upon CDDP treatment (Fig. 6g and h). These results suggested the pattern of miR-22 expression is depended on the intensity of the stresses in the presence of p53 activation.

## Discussions

Although significant advances have been made in the treatment of distinct types of cancer, drug resistance remains a major clinical obstacle. Better understanding of the underlying mechanisms is urgently needed to improve the current regimens of chemotherapy. In the present study, we showed for the first time that miR-22 expression level might be useful for predicting response to CDDP NAC in tongue cancer patients. High miR-22 expression in tongue cancer tissues strongly associated with increased sensitivity to CDDP regimen and high probability to achieve pCR, which is corroborated by the significant association between high miR-22 and better DFS (Fig. 1a and b). Otherwise, restored expression of miR-22 sensitized tongue cancer cells to CDDP, whereas functional inhibition of miR-22 led to enhanced resistance of tongue cancer cells. These results suggest that miR-22 plays a tumor suppressive role in tongue cancer.

It is well established that miRNAs can function as tumor suppressors or oncogenes depending on the cellular context and cancer types [32, 35, 36]. In tongue cancer, aberrant miRNA expression has been implicated to affect the response to various treatments, including chemotherapy, targeted therapies, and radiotherapy [18, 37, 38]. miR-22 plays unique roles in specific cell types via distinct mechanisms [39, 40]. For example, miR-22 represses the max expression to regulate the differentiation of a monocyte cell line [35], and it regulates the PPARα and BMP7 signaling pathways in human chondrocytes [[36]. The function of miR-22 in cancer seems controversial, and so far, most reports suggest that it is a tumor suppressor. miR-22 has been shown to inhibit proliferation and colony formation of MCF-7 cells by silencing c-Myc binding protein (MYCBP) [41] and to be downregulated in metastatic tongue cancer cells. Furthermore, the introduction of miR-22 reduced the expression of ERBB3 and EVI-1 (ectopic viral integration site-1) as well as phosphor-Akt, an EVI-1 target [42]. miR-22 downregulation was related to poor survival in hepatocellular carcinoma (HCC) patients, which may be due to the upregulation of histone deacetylase 4 (HDAC4), a potential miR-22 target [43]. On the other hand, miR-22 was reported to have an oncogenic role [44, 45]. The reported paradoxical role of miR-22 in cancer implies that miR-22 function is tissue-specific or context-dependent, and a comprehensive investigation is needed.

KAT6B is one of five members of the MYST HAT (histone acetyltransferases) family [46]. KAT6B has been shown to be transcriptional coregulator that physically and functionally interacts with the Runt-related transcription factor 1 (RUNX1), a recurrent leukemia-associated target, and the nuclear receptor peroxisome proliferator-activated receptor-α (PPARα) to exert its role [47, 48]. The KAT6B gene is rearranged in leukemia patients with t (10;16) (q22;p13) [23] and in leiomyoma cases with t (10;17) (p11;q21) [49]. The CBP gene is the fusion partner in the former translocation, while the GCN5 gene is a potential candidate in the latter translocation, suggesting that deregulated KAT6B has a vital role in oncogenesis. Here, we show that KAT6B is a direct target of miR-22, which may mediate miR-22-dependent chemosensitivity to cDDP regimen. Furthermore, we found that KAT6B levels in primary tumors of tongue cancer patients were inversely correlated with miR-22 expression, and the high expression of KAT6B was associated with poor prognosis (Fig. 4). In addition, miR-22 overexpression or KAT6B knockdown was accompanied by PI3K/Akt activity decrease, due to downregulation of S100A8, PDGF and VEGF. Although these secretory cytokines had been proved to activate PI3K/Akt pathway in previous researches [29–31], further studies are still needed to illuminates how KAT6B regulate these cytokine.

Different intensities of stress trigger different dominant signaling pathways to determine different cell fates. As shown in Fig. 6a-d, low-dose CDDP primarily triggered the survival signal, however, a lethal dose of CDDP specifically inhibited the survival signal, suggesting complex signaling in response to stress. In clinic treatment, the concentration of anti-cancer agents in different regions of a solid tumor is different and different signaling pathways are triggered, leading to the development of resistance in some cancer cells. The present study also provides a clue about when drug-resistance related factors selected by stress lead to resistance acquirement. In response to stress, the miR-22 expression pattern depended on the intensity of the stress. As shown here, high-dose CDDP induced miR-22 expression through p53 activation to promote cell death, and ectopic p53 expression also upregulated miR-22 expression. However, low-dose CDDP did not induced p53 activation or miR-22 expression. These results suggest that the pattern of miR-22 expression is depended on the intensity of the stresses in the presence of p53 activation.

Collectively, the intensity of the stress determined the cell fate in tongue cancer cells. High-dose stress mainly triggered the cell death signal; however, long-term low-dose exposure triggered a stronger survival signal than the death signal, leading to a new homeostasis with resistance to therapy. To some extent, this reflects the situation of solid cancer treatment in the clinic, where cells in different regions receive different drug concentrations, leading to the development of resistance. The present data imply a new potential strategy based on miR-22 combined with PI3K/Akt/NF-κB inhibition maintenance for tongue cancer chemotherapy or other cancers.

## Conclusions

Anticancer drug resistance is a major obstacle to successful cancer treatment, leading to cancer-related death. Our present study shows that miR-22 sensitizes primary tongue cancer cells to CDDP by targeting KAT6B expression. Furthermore, differences in the initial treatment stress determined the regulatory pattern of miR-22 expression. Exploring the molecular events in cancer cells upon administration of an anticancer drug will be valuable for fully understanding the mechanisms responsible for the acquisition and maintenance of drug resistance, which has been studied in the past decades. The results described here imply a potential strategy to clarify the mechanisms of resistance and delay or reverse drug resistance to improve the efficacy of cancer treatment.

## References

[1] Siegel R (2011). Cancer statistics, 2011: the impact of eliminating socioeconomic and racial disparities on premature cancer deaths. CA Cancer J Clin.

[2] Chen W (2016). Cancer statistics in China, 2015. CA Cancer J Clin.

[3] Pignon JP (2000). Chemotherapy added to locoregional treatment for head and neck squamous-cell carcinoma: three meta-analyses of updated individual data. MACH-NC collaborative group. Meta-analysis of chemotherapy on head and neck Cancer. Lancet.

[4] Seve P (2005). Expression of class III {beta}-tubulin is predictive of patient outcome in patients with non-small cell lung cancer receiving vinorelbine-based chemotherapy. Clin Cancer Res.

[5] Yamauchi K (2008). Induction of cancer metastasis by cyclophosphamide pretreatment of host mice: an opposite effect of chemotherapy. Cancer Res.

[6] Broxterman HJ, Gotink KJ, Verheul HM (2009). Understanding the causes of multidrug resistance in cancer: a comparison of doxorubicin and sunitinib. Drug Resist Updat.

[7] Sarkar FH (2010). Implication of microRNAs in drug resistance for designing novel cancer therapy. Drug Resist Updat.

[8] Croce CM, Calin GA (2005). miRNAs, cancer, and stem cell division. Cell.

[9] Croce CM (2009). Causes and consequences of microRNA dysregulation in cancer. Nat Rev Genet.

[10] Blower PE (2008). MicroRNAs modulate the chemosensitivity of tumor cells. Mol Cancer Ther.

[11] Fujita Y (2008). Effects of miR-34a on cell growth and chemoresistance in prostate cancer PC3 cells. Biochem Biophys Res Commun.

[12] Rui W (2010). Identification of microRNA profiles in docetaxel-resistant human non-small cell lung carcinoma cells (SPC-A1). J Cell Mol Med.

[13] Adam L (2009). miR-200 expression regulates epithelial-to-mesenchymal transition in bladder cancer cells and reverses resistance to epidermal growth factor receptor therapy. Clin Cancer Res.

[14] Li Y (2009). Up-regulation of miR-200 and let-7 by natural agents leads to the reversal of epithelial-to-mesenchymal transition in gemcitabine-resistant pancreatic cancer cells. Cancer Res.

[15] Wong TS (2008). Mature miR-184 as potential oncogenic microRNA of squamous cell carcinoma of tongue. Clin Cancer Res.

[16] Li J (2009). MiR-21 indicates poor prognosis in tongue squamous cell carcinomas as an apoptosis inhibitor. Clin Cancer Res.

[17] Qiu K (2016). miR-22 regulates cell invasion, migration and proliferation in vitro through inhibiting CD147 expression in tongue squamous cell carcinoma. Arch Oral Biol.

[18] Sun L (2012). MiR-200b and miR-15b regulate chemotherapy-induced epithelial-mesenchymal transition in human tongue cancer cells by targeting BMI1. Oncogene.

[19] Seligson DB (2005). Global histone modification patterns predict risk of prostate cancer recurrence. Nature.

[20] Fraga MF (2005). Loss of acetylation at Lys16 and trimethylation at Lys20 of histone H4 is a common hallmark of human cancer. Nat Genet.

[21] Berdasco M, Esteller M (2010). Aberrant epigenetic landscape in cancer: how cellular identity goes awry. Dev Cell.

[22] Dawson MA, Kouzarides T (2012). Cancer epigenetics: from mechanism to therapy. Cell.

[23] Panagopoulos I (2001). Fusion of the MORF and CBP genes in acute myeloid leukemia with the t(10,16)(q22;p13). Hum Mol Genet.

[24] Ohta K (2007). Histone acetyltransferase MOZ acts as a co-activator of Nrf2-MafK and induces tumour marker gene expression during hepatocarcinogenesis. Biochem J.

[25] Fong MY (2015). Breast-cancer-secreted miR-122 reprograms glucose metabolism in premetastatic niche to promote metastasis. Nat Cell Biol.

[26] Chen QX (2015). A general approach to high-yield biosynthesis of chimeric RNAs bearing various types of functional small RNAs for broad applications. Nucleic Acids Res.

[27] Li MM (2015). Chimeric MicroRNA-1291 biosynthesized efficiently in Escherichia coli is effective to reduce target gene expression in human carcinoma cells and improve Chemosensitivity. Drug Metab Dispos.

[28] Gu Y (2017). Epigenetic silencing of miR-493 increases the resistance to cisplatin in lung cancer by targeting tongue cancer resistance-related protein 1(TCRP1). J Exp Clin Cancer Res.

[29] Ghavami S (2009). S100A8/A9: a Janus-faced molecule in cancer therapy and tumorgenesis. Eur J Pharmacol.

[30] Chan CM (2013). Inhibitory effects of resveratrol on PDGF-BB-induced retinal pigment epithelial cell migration via PDGFRbeta, PI3K/Akt and MAPK pathways. PLoS One.

[31] Ruan GX, Kazlauskas A (2012). VEGF-A engages at least three tyrosine kinases to activate PI3K/Akt. Cell Cycle.

[32] Tsuchiya N (2011). Tumor suppressor miR-22 determines p53-dependent cellular fate through post-transcriptional regulation of p21. Cancer Res.

[33] Green DR, Kroemer G (2009). Cytoplasmic functions of the tumour suppressor p53. Nature.

[34] Petitjean A (2007). TP53 mutations in human cancers: functional selection and impact on cancer prognosis and outcomes. Oncogene.

[35] Ting Y (2010). Differentiation-associated miR-22 represses max expression and inhibits cell cycle progression. Biochem Biophys Res Commun.

[36] Iliopoulos D (2008). Integrative microRNA and proteomic approaches identify novel osteoarthritis genes and their collaborative metabolic and inflammatory networks. PLoS One.

[37] Lin Z (2014). miR-639 regulates transforming growth factor beta-induced epithelial-mesenchymal transition in human tongue cancer cells by targeting FOXC1. Cancer Sci.

[38] Zhou XL (2015). Integrated microRNA-mRNA analysis revealing the potential roles of microRNAs in tongue squamous cell cancer. Mol Med Rep.

[39] Chang TC (2008). Widespread microRNA repression by Myc contributes to tumorigenesis. Nat Genet.

[40] Cornelis RS (1994). Evidence for a gene on 17p13.3, distal to TP53, as a target for allele loss in breast tumors without p53 mutations. Cancer Res.

[41] Xiong J, Du Q, Liang Z (2010). Tumor-suppressive microRNA-22 inhibits the transcription of E-box-containing c-Myc target genes by silencing c-Myc binding protein. Oncogene.

[42] Patel JB (2011). Control of EVI-1 oncogene expression in metastatic breast cancer cells through microRNA miR-22. Oncogene.

[43] Zhang J (2010). microRNA-22, downregulated in hepatocellular carcinoma and correlated with prognosis, suppresses cell proliferation and tumourigenicity. Br J Cancer.

[44] Bar N, Dikstein R (2010). miR-22 forms a regulatory loop in PTEN/AKT pathway and modulates signaling kinetics. PLoS One.

[45] Tan G, Shi Y, Wu ZH (2012). MicroRNA-22 promotes cell survival upon UV radiation by repressing PTEN. Biochem Biophys Res Commun.

[46] Sterner DE, Berger SL (2000). Acetylation of histones and transcription-related factors. Microbiol Mol Biol Rev.

[47] Pelletier N (2002). MOZ and MORF histone acetyltransferases interact with the runt-domain transcription factor Runx2. Oncogene.

[48] Surapureddi S (2002). Identification of a transcriptionally active peroxisome proliferator-activated receptor alpha -interacting cofactor complex in rat liver and characterization of PRIC285 as a coactivator. Proc Natl Acad Sci U S A.

[49] Moore SD (2004). Uterine leiomyomata with t(10,17) disrupt the histone acetyltransferase MORF. Cancer Res.

